# Supplementary material for: Statin prevents cancer development in chronic inflammation by blocking interleukin 33 expression
Source: Nat Commun. 2024 May 30;15:4099. doi: 10.1038/s41467-024-48441-8 (PMC11139893; doi:10.1038/s41467-024-48441-8)
Supplement: Supplementary file 1 — Supplementary Information [file 41467_2024_48441_MOESM1_ESM.pdf]

Supplementary Information

Statin prevents cancer development in chronic inflammation by blocking interleukin 33 expression

Jong Ho Park[1,2,10], Mahsa Mortaja[1,2], Heehwa G. Son[1,2], Xutu Zhao[1,2], Lauren M. Sloat[1,2], Marjan Azin[1,2], Jun Wang[2], Michael R. Collier[3], Krishna S. Tummala[4-7,11], Anna Mandinova[2], Nabeel Bardeesy[4-7], Yevgeniy R. Semenov[3,8], Mari Mino-Kenudson[9], and Shadmehr Demehri[1,2,3*]

[1]Center for Cancer Immunology, Krantz Family Center for Cancer Research, Massachusetts General Hospital and Harvard Medical School, Boston, MA, USA
[2]Cutaneous Biology Research Center, Department of Dermatology, Massachusetts General Hospital and Harvard Medical School, Boston, MA, USA
[3]Department of Dermatology, Massachusetts General Hospital and Harvard Medical School, Boston, MA, USA
[4]Krantz Family Center for Cancer Research, Massachusetts General Hospital and Harvard Medical School, Boston, MA, USA
[5]Center for Regenerative Medicine, Massachusetts General Hospital, Boston MA, USA
[6]Department of Medicine, Harvard Medical School, Boston, MA, USA
[7]Cancer Program, Broad Institute of Massachusetts Institute of Technology and Harvard, Cambridge, MA, USA
[8]Laboratory of Systems Pharmacology, Harvard Program in Therapeutic Science, Harvard Medical School, Boston, USA
[9]Department of Pathology, Massachusetts General Hospital and Harvard Medical School, Boston, MA, USA
[10]Department of Anatomy, School of Medicine, Keimyung University, Daegu, South Korea
[11]Quantitative Biosciences, Merck Research Laboratories, Boston, MA, USA

* Author for correspondence:
Shadmehr Demehri, M.D., Ph.D.
Department of Dermatology and MGH Cancer Center
Building 149 13th Street, 3rd floor
Charlestown MA 02129
Phone: 617-643-6436, Fax: 617-726-4453
Email: sdemehri1@mgh.harvard.edu

Supplementary Figures

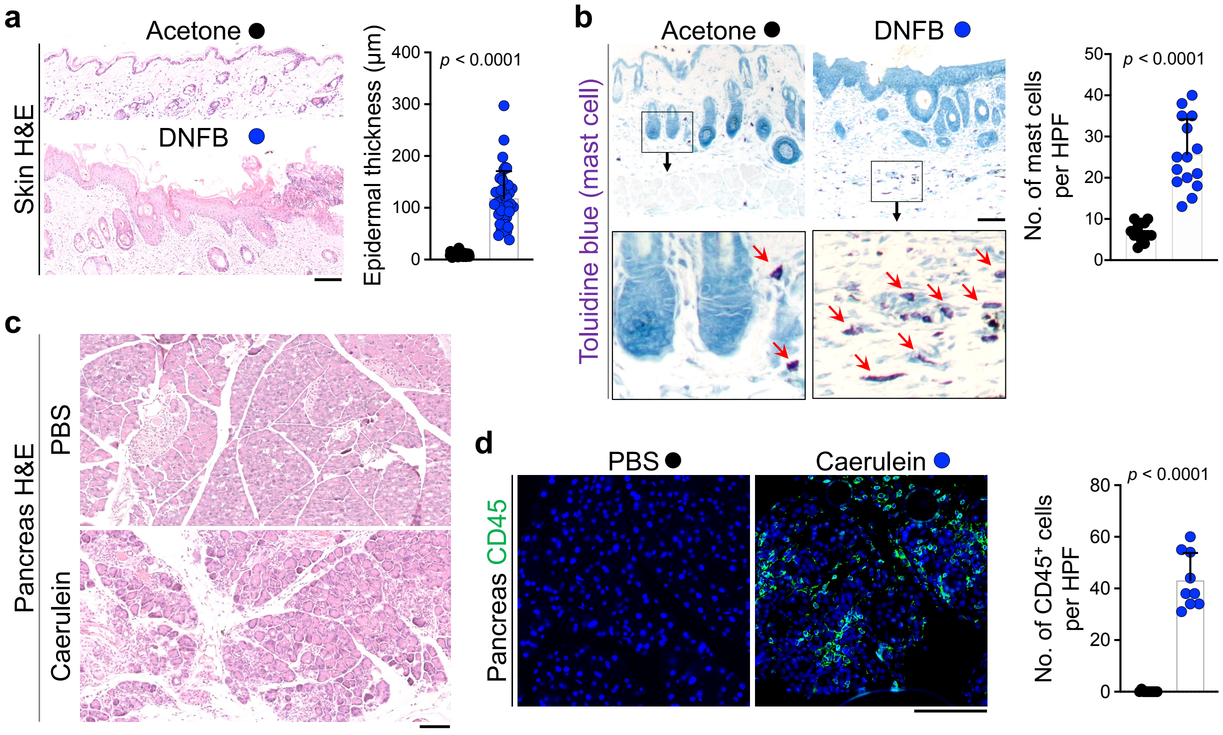

Supplementary Fig. 1 | Induction of chronic inflammation in the skin and pancreas. a (Left) Representative images of H&E-stained skin of DNFB- and acetone-treated WT mice. (Right) The epidermal thickness of DNFB- and acetone-treated WT skin. Each dot represents the average of three measurements in a high-power field (HPF) image. Ten random HPF images per skin sample are included (*n*=4 mice in each group). **b** (Left) Representative images of toluidine blue-stained skin of DNFB- and acetone-treated WT mice. Red arrows in insets highlight mast cells in purple. (Right) Quantification of mast cells in DNFB- and acetone-treated WT skin. Each dot represents cell counts from an HPF image. Five randomly selected HPF images are included per skin sample (*n*=3 mice in each group).**c** Representative images of H&E-stained pancreas of caerulein- and PBS-treated WT mice. **d** (Left) Representative images of CD45-stained pancreas of caerulein- and PBS-treated WT mice. (Right) $CD45^+$ immune cell counts in caerulein- and PBS-treated WT pancreas. Each dot represents cell counts from an HPF image. Three randomly selected HPF

images are included per pancreas sample (*n*=3 mice in each group). Graphs show mean + SD, two-sided unpaired *t*-test, scale bars: 100 µm. Source data are provided as a Source Data file.

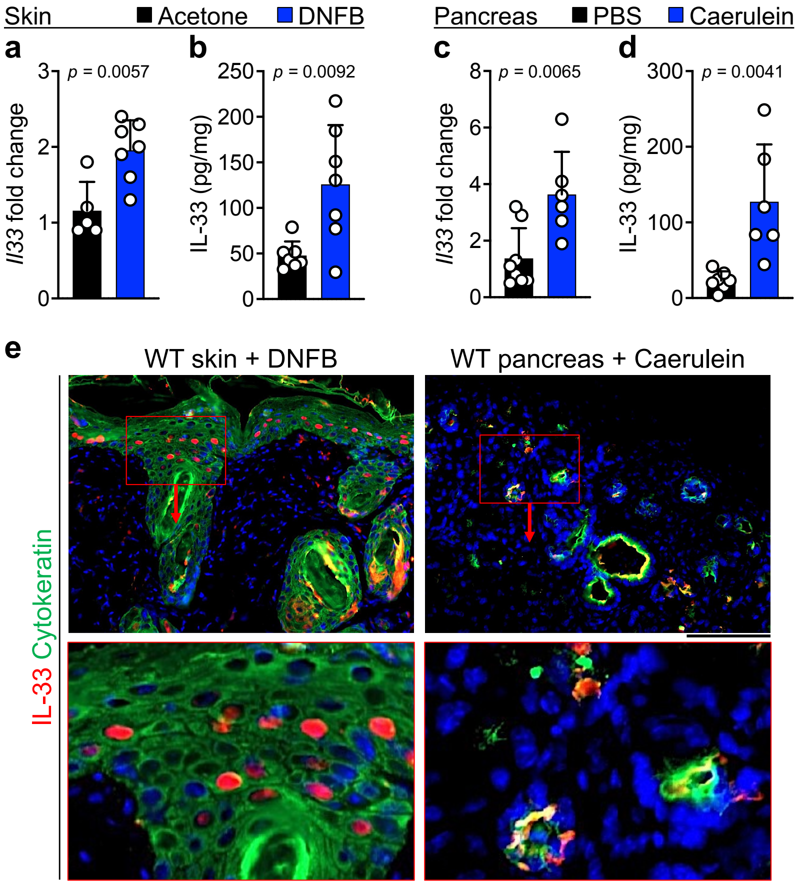

f

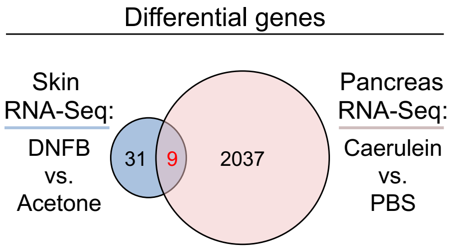

g

Gene name	**Skin RNA-Seq**		**Pancreas RNA-Seq**	
	Fold change (log_2)	Adj *p*-value	Fold change (log_2)	Adj *p*-value
S100a9	5.85902	0.004449	3.047692	0.004716
S100a8	5.332878	0.00017	6.457228	0.002023
Acod1	5.276973	0.000451	4.864986	0.000525
Spp1	4.490095	0.000676	4.168989	0.002719
Srgn	3.18926	0.000271	5.481803	0.002709
Cxcl2	3.098742	0.033914	1.373108	0.017939
Glrx	2.065748	0.022658	1.726516	0.003977
Hdc	0.202533	0.004061	3.584037	0.002719
Slpi	0.155427	0.004061	5.088557	0.000651

Supplementary Fig. 2 | IL-33 induction is associated with DAMPs upregulation in chronic inflammation. a *Il33* expression in DNFB- versus acetone-treated WT skin (*n*=7 mice in DNFB and *n*=5 mice in acetone group). **b** IL-33 protein levels in DNFB- versus acetone-treated WT skin (*n*=7 mice in each group). **c** *Il33* expression in caerulein- versus PBS-treated WT pancreas (*n*=6

mice in caerulein and *n*=8 mice in PBS group). **d** IL-33 protein levels in caerulein- versus PBS-treated WT pancreas (*n*=6 mice in caerulein and *n*=7 mice in PBS group). **e** Representative images of IL-33 and cytokeratin-stained inflamed skin and pancreas. **f** Venn diagram of shared differentially expressed genes from RNA sequencing (RNA-Seq) of the DNFB-treated skin and caerulein-treated pancreas compared with acetone- and PBS-treated controls, respectively. **g** The list of nine differentially expressed genes shared between chronically inflamed skin and pancreas. Graphs show mean + SD, two-sided unpaired *t*-test, scale bar: 100 µm. Source data are provided as a Source Data file.

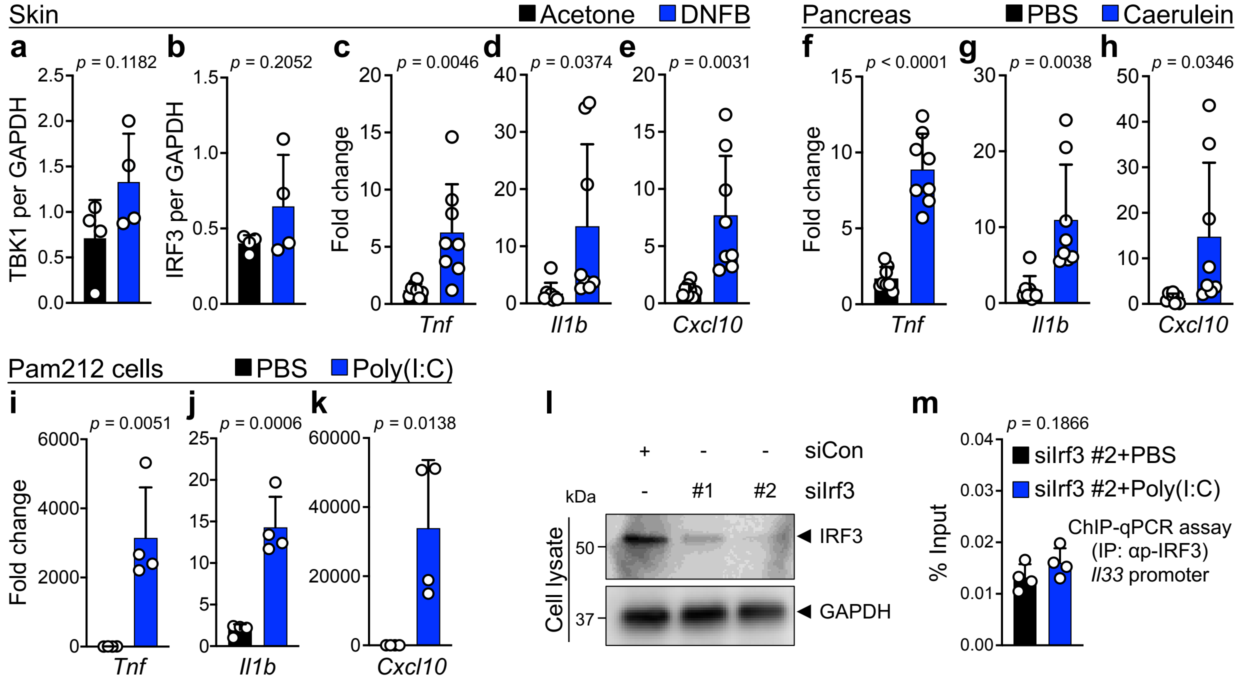

Supplementary Fig. 3 | IRF3 target genes are upregulated in chronic inflammation. a, b The ratio of TBK1/GAPDH and IRF3/GAPDH protein band intensity quantified from the immunoblot is shown in Fig.1d. **c-e** *Tnf* (c), *Il1b* (d), and *Cxcl10* (e) expression in DNFB- versus acetone-treated WT skin (*n*=8 mice in each group). **f-h** *Tnf* (f), *Il1b* (g), and *Cxcl10* (h) expression in caerulein- versus PBS-treated WT pancreas (*n*=8 mice in each group). **i-k,** *Tnf* (i), *Il1b* (j), and *Cxcl10* (k) expression in poly(I:C) versus PBS-treated Pam212 cells (*n*=4 cell culture plates in each group). **l** Immunoblot of IRF3 and GAPDH proteins in siIrf3- versus siCon-treated Pam212 cells. **m** ChIP-qPCR assay for p-IRF3 binding to Il33 promoter region after siIrf3-treated Pam212 cells exposed to poly(I:C) versus PBS (*n*=4 cell culture plates in each group). Graphs show mean + SD, two-sided unpaired *t*-test. Source data are provided as a Source Data file.

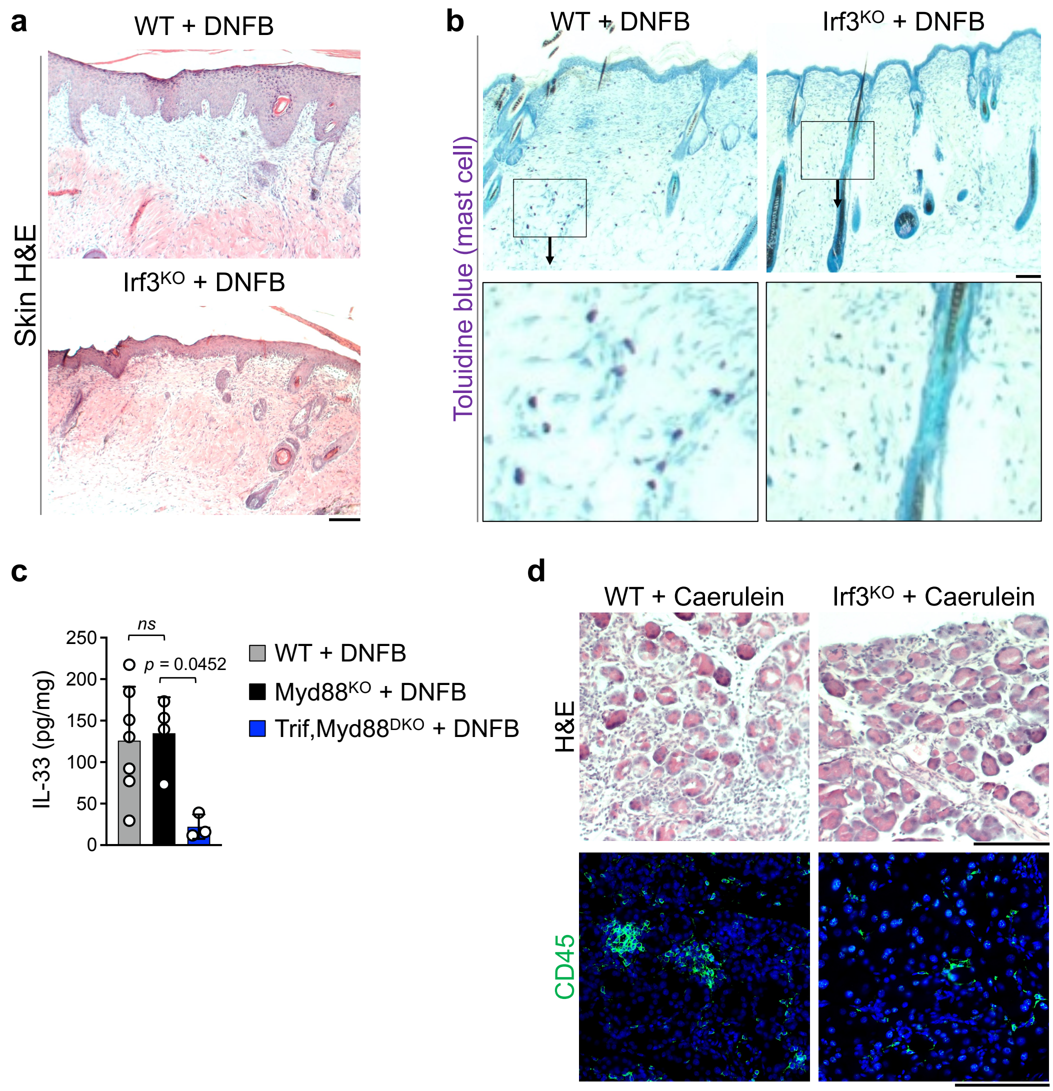

Supplementary Fig. 4 | IRF3 is required for the development of chronic inflammation. a Representative images of H&E-stained skin of DNFB-treated WT or Irf3^{KO} mice. **b** Representative images of toluidine blue-stained skin of DNFB-treated WT or Irf3^{KO} mice. Insets highlight the dense collection of mast cells (purple) in WT skin compared with infrequent mast cells in Irf3^{KO} skin. **c** IL-33 protein levels in DNFB-treated WT, Myd88^{KO} and Trif,Myd88^{DKO} skin (n=7 mice in WT, n=4 mice in Myd88^{KO} and n=3 mice in Trif,Myd88^{DKO} group). WT group values are also shown in Supplementary Fig. 2b. **d** Representative images of H&E- and CD45-stained pancreas of

caerulein-treated WT and Irf3^{KO} mice. Graphs show mean + SD, two-sided unpaired *t*-test, scale bars: 100 µm. Source data are provided as a Source Data file.

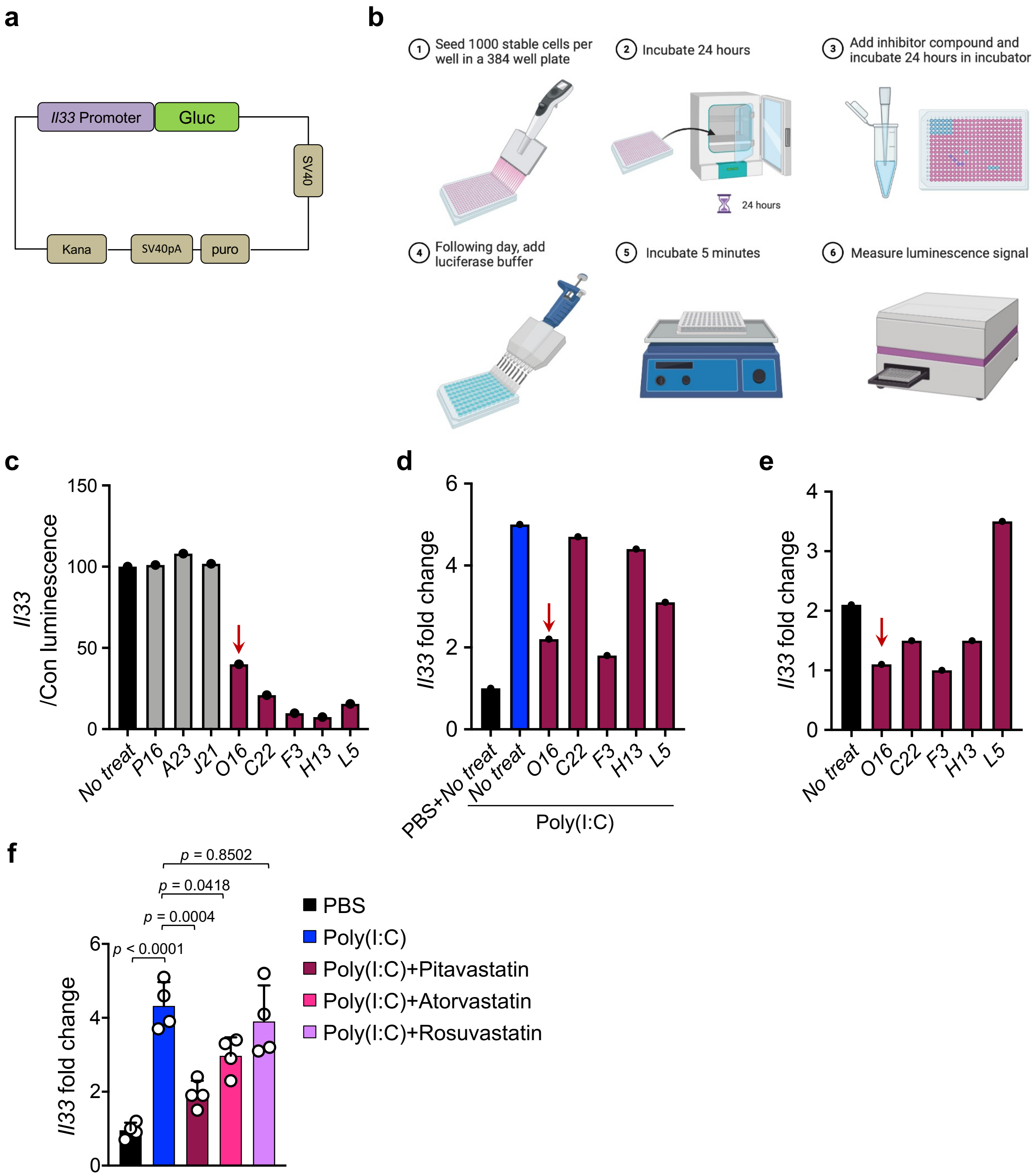

Supplementary Fig. 5 | Small molecule IL-33 inhibitor discovery platform. **a** Schematic of *Il33* gene promoter vector constructs for an FDA-approved drug library screening. **b** Schematic method of luciferase screening assay. **c** Candidate compounds with inhibitory effect on *Il33* reporter (created with BioRender.com). **d** Candidate inhibitory compounds' effect on *Il33* expression induced by poly(I:C) in Pam212 cells. The red arrow points to pitavastatin calcium

(labeled as O16 in the screening process). The first “no treat” condition was treated with PBS, and the second “no treat” was treated with poly (I:C) without small molecules. **e** Inhibitory effect of 5 candidate drugs on endogenous *Il33* expression in $PyMt^{tg}$ breast cancer cell line, which has high endogenous expression of IL-33. **f** *Il33* expression in poly(I:C)-treated Pam 212 cells that received pitavastatin, atorvastatin versus rosuvastatin (*n*=4 cell culture plates in each group). Graphs show mean + SD, one-way ANOVA. Source data are provided as a Source Data file.

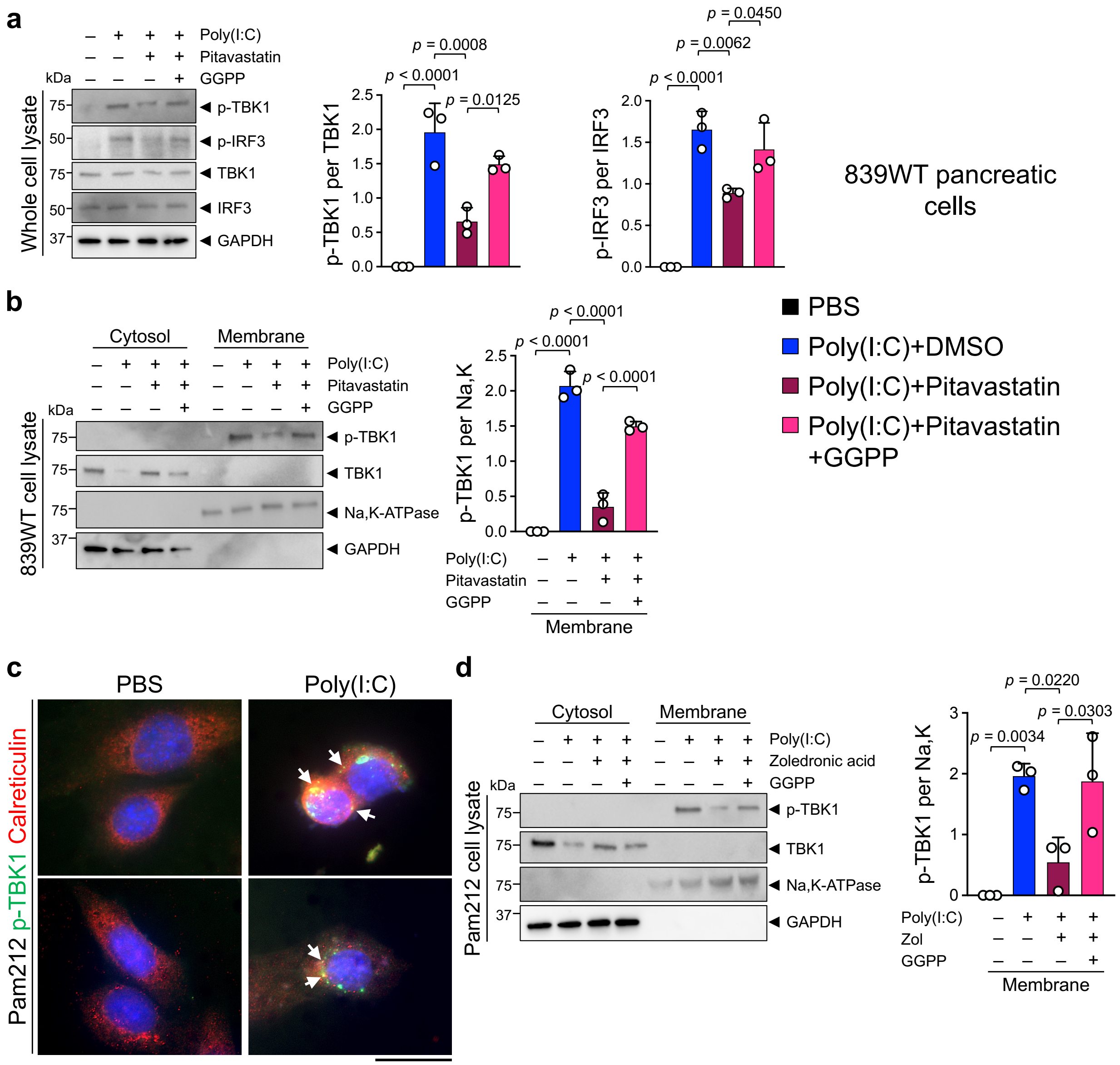

Supplementary Fig. 6 | Mevalonate pathway inhibition blocks TBK1 signaling in a pancreatic cell line, which is associated with reduced membrane-bound p-TBK1. a (Left) Immunoblot of p-TBK1, p-IRF3, TBK1, IRF3, and GAPDH proteins in whole cell lysates of PBS- versus poly(I:C)-treated 839WT pancreatic cells that received pitavastatin (10 μM) alone or in combination with GGPP (3 μM) (*n*=3 cell culture plates in each group). Cells were harvested after 6-hour incubation with poly(I:C), pitavastatin, and GGPP. (Right) The ratio of p-TBK1/TBK1 and p-IRF3/IRF3 protein band intensity from immunoblots (*n*=3 cell culture plates in each group). **b**

(Left) Immunoblot of p-TBK1, TBK1, Na,K-ATPase, and GAPDH proteins in membrane and cytosol fraction of poly(I:C)-treated 839WT cells that received pitavastatin (10 μM) alone or in combination with GGPP (3 μM). Cells were harvested after 6-hour incubation with poly(I:C), pitavastatin, and GGPP. (Right) The ratio of membrane-bound p-TBK1/Na,K-ATPase protein band intensity from the immunoblots (*n*=3 cell culture plates in each group). **c** Representative images of p-TBK1 and calreticulin-stained poly(I:C)- versus PBS-treated Pam212 cells. Arrows point to the co-localization of p-TBK1 and calreticulin in the cells. **d** (Left) Immunoblot of p-TBK1, TBK1, Na,K-ATPase, and GAPDH proteins in membrane and cytosol faction of poly(I:C)-treated Pam212 cells that received zoledronic acid (Zol, 10 μM) alone or in combination with GGPP (3 μM). Cells were harvested after 6-hour incubation with poly(I:C), zoledronic acid, and GGPP. (Right) The ratio of membrane-bound p-TBK1 to Na,K-ATPase protein band intensity from the immunoblots (*n*=3 cell culture plates in each group). Graphs show mean + SD, one-way ANOVA, scale bar: 25 μm. Source data are provided as a Source Data file.

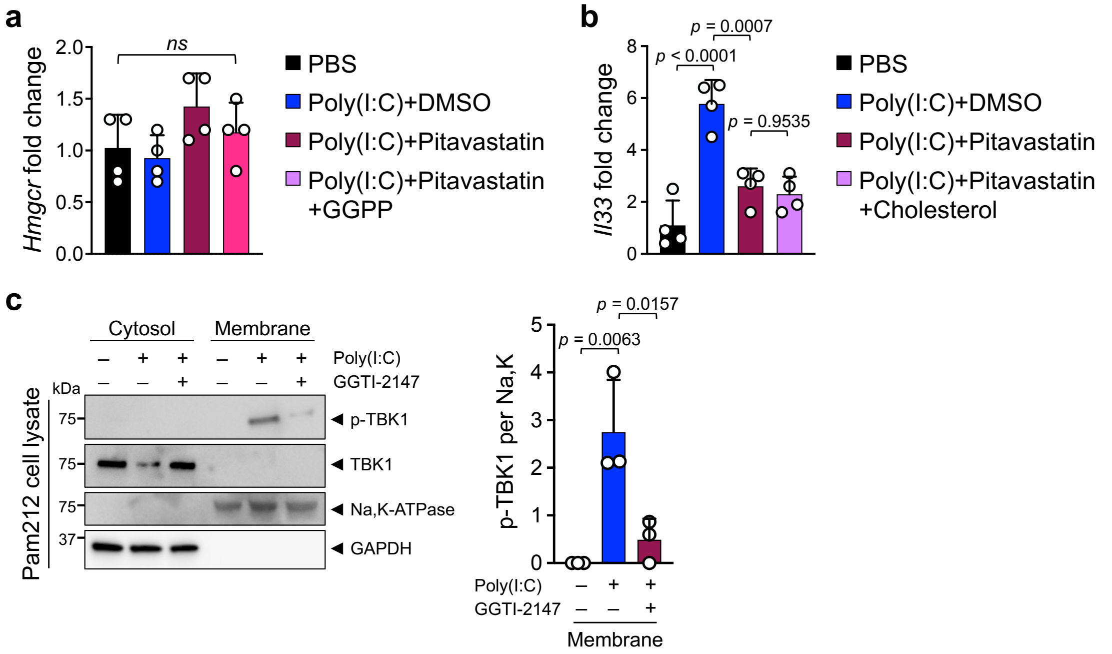

Supplementary Fig. 7 | Cholesterol level does not impact the effect of pitavastatin on TBK1-IL-33 signaling. a *Hmgcr* expression in PBS- versus poly(I:C)-treated Pam212 cells that received pitavastatin (10 μM) alone or in combination with GGPP (3 μM) (*n*=4 cell culture plates in each group). Cells were harvested after 6-hour incubation with poly(I:C), pitavastatin, and GGPP. **b** *Il33* expression in PBS- versus poly(I:C)-treated Pam212 cells that received pitavastatin (10 μM) alone or in combination with cholesterol (5 μg/mL) (*n*=4 cell culture plates in each group). Cells were harvested after 6-hour incubation with poly(I:C), pitavastatin, and cholesterol. **c** (Left) Representative immunoblot of p-TBK1, TBK1, Na.K-ATPase and GAPDH proteins in membrane and cytosol fraction of poly(I:C)-treated Pam212 cells +/- geranylgeranyltransferase I (GGTase-I) inhibitor (GGTI-2147, 5 μM). Cells were harvested after 6-hour incubation with poly(I:C), pitavastatin, and GGTI-2147. (Right) The ratio of membrane-bound p-TKB1 to Na,K-ATPase protein band intensity from the immunoblot (*n*=3 cell culture plates in each group). Graphs show mean + SD, one-way ANOVA. Source data are provided as a Source Data file.

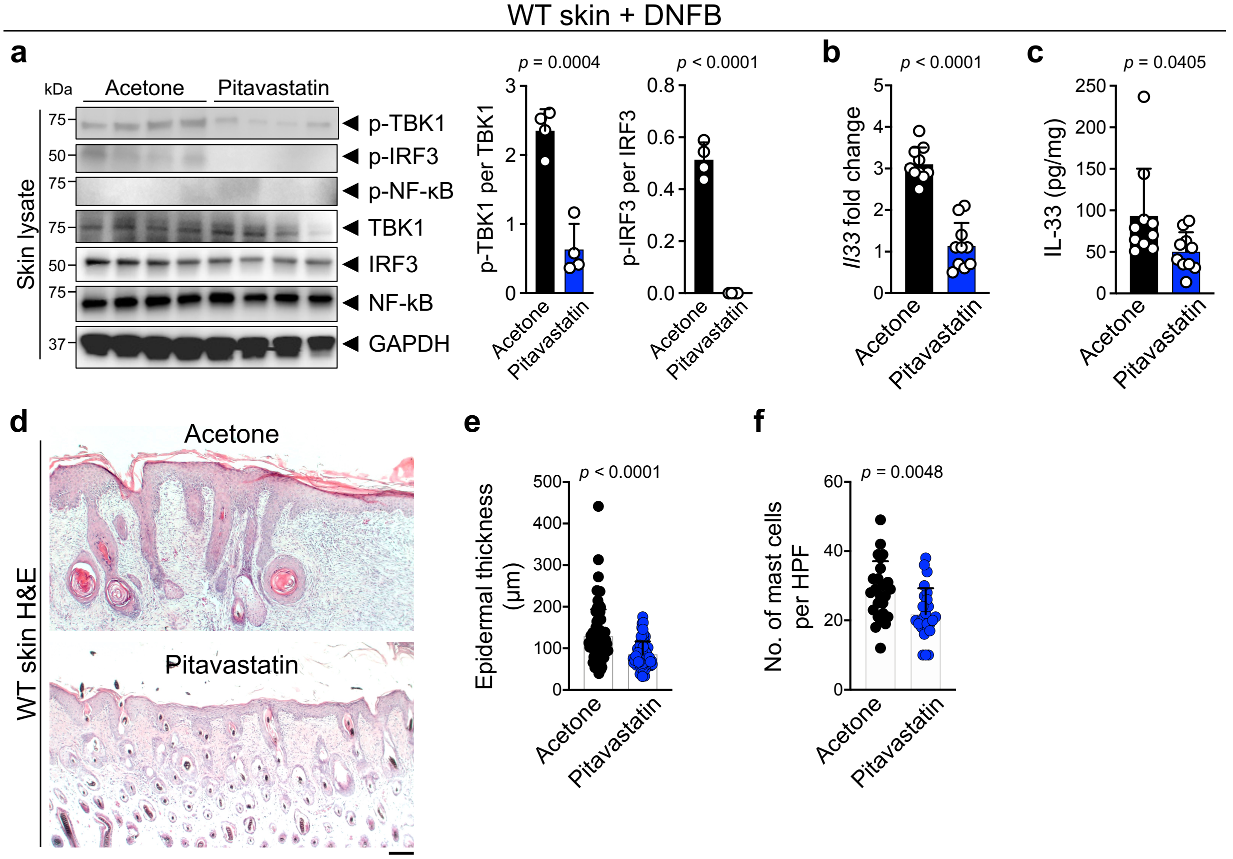

Supplementary Fig. 8 | Pitavastatin blocks chronic inflammation in the skin. a (Left) Immunoblot of p-TBK1, p-IRF3, p-NF-κB, TBK1, IRF3, NF-κB, and GAPDH proteins in topical pitavastatin- versus acetone-treated WT skin at the completion of the DNFB treatment protocol (*n*=4 mice in each group). (Right) The ratio of p-TBK1/TBK1 and p-IRF3/IRF3 protein band intensity quantified from the immunoblot shown on the left. **b** *Il33* expression in pitavastatin- versus acetone-treated WT skin at the completion of DNFB treatment protocol (*n*=10 mice in each group). **c** IL-33 protein levels in pitavastatin- versus acetone-treated WT skin at the completion of DNFB treatment protocol (*n*=10 mice in each group). **d** Representative images of H&E-stained skin from pitavastatin- and acetone-treated WT mice at the completion of the DNFB treatment protocol. **e** Epidermal thickness of pitavastatin- versus acetone-treated WT skin at the completion of DNFB treatment protocol. Each dot represents the average of three measurements in an HPF image. Ten random HPF images per skin sample are included (*n*=8 mice in each group). **f** Mast

cell counts in pitavastatin- versus acetone-treated WT skin at the completion of DNFB treatment protocol. Each dot represents cell counts from an HPF image. Five randomly selected HPF images are included per sample (*n*=5 mice in each group). Graphs show mean + SD, two-sided unpaired *t*-test, scale bar: 100 µm. Source data are provided as a Source Data file.

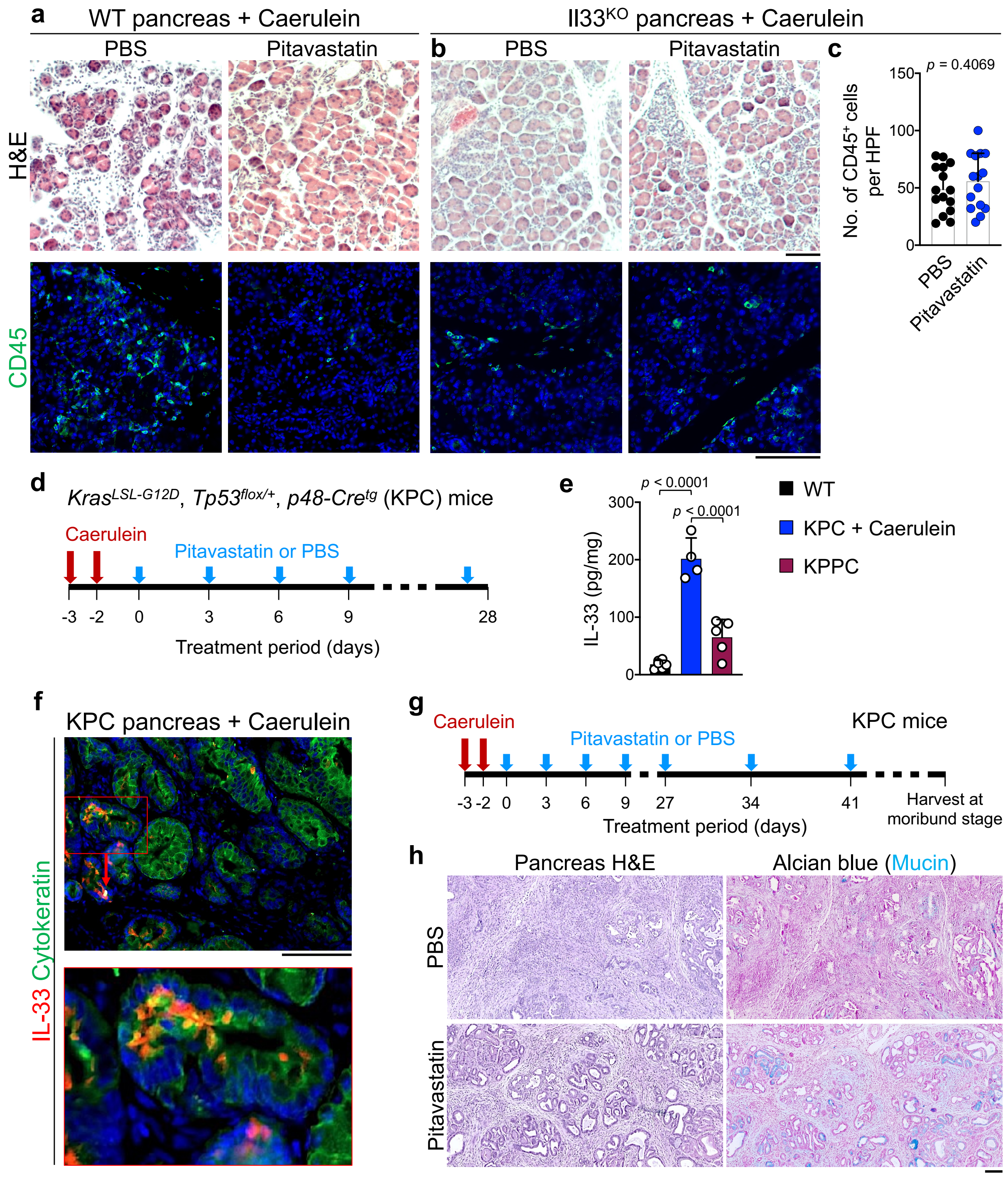

Supplementary Fig. 9 | Pitavastatin blocks chronic pancreatitis and its cancer sequela in an IL-33-dependent manner. a Representative images of H&E- and CD45-stained pancreas from pitavastatin- and PBS-treated WT mice at the completion of the caerulein treatment protocol. **b** Representative images of H&E- and CD45-stained pancreas from pitavastatin- and PBS-treated

$Il33^{KO}$ mice at the completion of the caerulein treatment protocol. **c** $CD45^+$ immune cell counts in pitavastatin- and PBS-treated $Il33^{KO}$ pancreas at the completion of the caerulein treatment protocol. Each dot represents cell counts from an HPF image. Three randomly selected HPF images are included per mouse pancreas (*n*=5 mice in each group). **d** Schematic diagram of the experimental design for the induction of chronic inflammation-associated pancreas cancer. KPC mice received seven hourly intraperitoneal caerulein injections over two days, followed by intraperitoneal pitavastatin versus PBS injections once every three days over four weeks. **e** IL-33 protein levels in WT, caerulein-treated KPC, and KPPC (*Tp53* homogenous floxed mouse) (*n*=6 mice in WT, *n*=4 mice in caerulein-treated KPC, and *n*=5 mice in KPPC group). **f** Representative images of IL-33 and cytokeratin-stained pancreatic cancer in caerulein-treated KPC mice. **g** Schematic diagram of the experimental design for the induction of chronic inflammation-associated pancreas cancer in mice for survival analysis. KPC mice received seven hourly intraperitoneal caerulein injections over two days. Next, mice received intraperitoneal pitavastatin versus PBS injections once every three days over four weeks, followed by weekly treatments until the animal reached a moribund stage. **h** Representative images of H&E and Alcian blue-stained terminal pancreatic tumors from pitavastatin- versus PBS-treated KPC mice that underwent caerulein-induced pancreatic cancer protocol. Graphs show mean + SD, two-sided unpaired *t*-test (c), one-way ANOVA (e), scale bars: 100 μm. Source data are provided as a Source Data file.

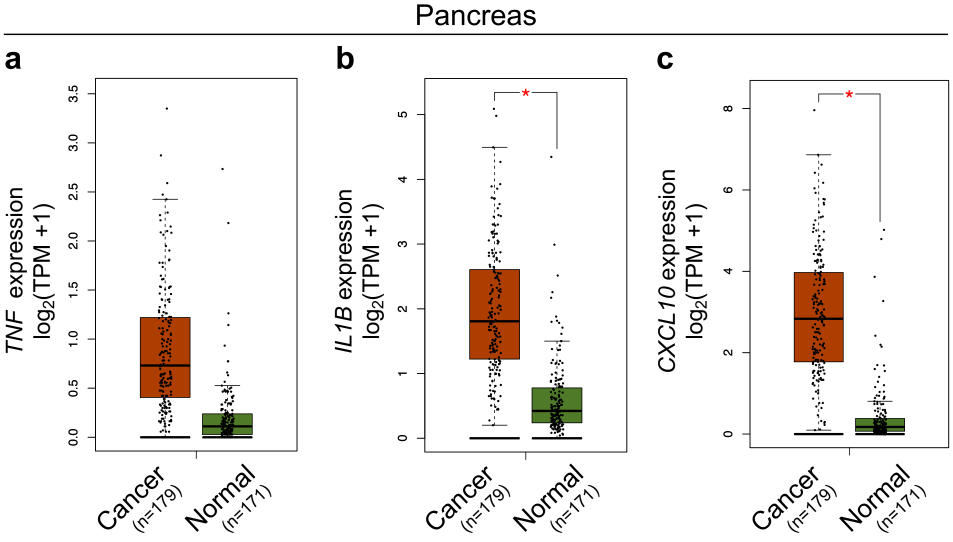

Supplementary Fig. 10 | IRF3 target genes are upregulated in human pancreatic cancer. a-c Box plot of *TNF* (a), *IL1B* (b), and *CXCL10* (c) expression in pancreatic cancer versus normal pancreas across TCGA/GTEx datasets (* $p < 0.01$, one-way ANOVA, Gene Expression Profiling Interactive Analysis database). Box plots show median (center line), interquartile range (box limits), 1.5 times the interquartile range (whiskers length), and outliers (points outside whiskers).

Supplementary Tables

Supplementary Table 1. Characteristics of pitavastatin and ezetimibe treatment cohorts.

**Baseline Characteristics: Matched Cohorts**				
	Pitavastatin		Ezetimibe	
Number of patients	242,645		242,645	
Age at index (years)	Mean	SD	Mean	SD
	63.8	10.9	63.4	11.7
Gender	N	%	N	%
Male	102,968	42.436	101,989	42.032
Female	139,588	57.528	140,591	57.941
Unknown	89	0.037	65	0.027
Race	Mean	%	Mean	%
White	79,571	32.793	76,501	31.528
Black	6,103	2.515	5,829	2.402
Asian	1,624	0.669	876	0.361
American Indian or	0	0	0	0
Native Hawaiian and	0	0	0	0
Unknown	155,347	64.022	159,439	65.709
Ethnicity	Mean	%	Mean	%
Hispanic or Latino	4,433	1.827	4,500	1.855
Not Hispanic or Latino	84,913	34.995	80,960	33.366
Unknown	153,299	63.178	157,185	64.78

Supplementary Table 2. Antibodies, staining kits, and primers information.

Western blotting Antibodies	Clone	Company	Dilution factor
Primary antibodies			
p-TBK1 (#5483)	D52C2	Cell Signaling Technology, Danvers, MA	1:1000
p-IRF3 (#29047)	D601M	Cell Signaling Technology	1:1000
p-NF-κB (#3033)	93H1	Cell Signaling Technology	1:1000
TBK1 (#3504)	D1B4	Cell Signaling Technology	1:1000
IRF3 (#4302)	D83B9	Cell Signaling Technology	1:1000
GAPDH (#5174)	D16H11	Cell Signaling Technology	1:1000
Anti-mouse IL-33 (#AF3626)	Polyclonal	R&D Systems, Minneapolis, MN	0.4ug/mL
NF-κB (#8242)	D14E12	Cell Signaling Technology	1:1000
Na,K-ATPase (#3010)	Polyclonal	Cell Signaling Technology	1:1000

Secondary antibodies	Cat #	Company	Dilution factor
Peroxidase Goat Anti-Mouse IgG	115-035-003	Jackson ImmunoResearch, West Grove, PA	1:5000
Peroxidase Goat Anti-Rabbit IgG	111-035-003	Jackson ImmunoResearch	1:5000

siRNA	Cat #	Company
siTrif (siTicam1)	4390771	Thermo Fisher Scientific, Waltham, MA
siIrf3	SI00210770	QIAGEN, Hilden, Germany
siCon	1022076	QIAGEN

Immunohistochemical staining antibodies	Clone	Company	Dilution factor
IL-33 (mouse) (#ALX804840C100)	Nessy-1	ENZO, Farmingdale, NY	1:200
IL-33 (human) (#HPA024426)	Polyclonal	Sigma, St. Louis, MO	1:200
IRF3 (human) (#SC-33641)	SL-12	Santa Cruz Biotechnology, Dallas, TX	1:50

Staining kits	Cat #	Company
Antigen unmasking solution	H3300	Vector Laboratories, Burlingame, CA
VECTASTAIN Elite ABC universal Kit	PK6200	Vector Laboratories
ImmPACT DAB chromogen staining kit	SK-4105	Vector Laboratories
Alexa Fluor 594 Tyramide superboost kit, goat anti-mouse	B40942	Thermo Fisher Scientific
Alexa Fluor 488 Tyramide superboost kit, goat anti-rabbit	B40943	Thermo Fisher Scientific

Immunofluorescence staining antibodies	**Clone**	**Company**	**Dilution factor**
CD45 (#AB10558)		Abcam, Waltham, MA	1:500
Cytokeratin (mouse) (#M3515)	AE1/AE3	Agilent Dako, Santa Clara, CA	1:100
IL-33 (mouse) (#ALX804840C100)	Nessy-1	ENZO	1:200
p-TBK1 (#5483)	D52C2	Cell Signaling Technology	1:100
Calreticulin (#12238)	D3E6	Cell Signaling Technology	1:200

Secondary antibodies	**Cat #**	**Company**	**Dilution factor**
Goat anti-Rabbit IgG, Alexa Fluor 488 conjugate	A11034	Thermo Fisher Scientific, Waltham, MA	1:200
Goat anti-Rabbit IgG, Alexa Fluor 568 conjugate	A11036	Thermo Fisher Scientific	1:200
Goat anti-mouse IgG(H+L), AF488	1031-30	SouthernBiotech, Birmingham, AL	1:200

**qPCR primers**	**Cat #**	**Company**
Taqman		
Il33	Mm.PT.58. 12022572	Integrated DNA Technologies, Coralville, Iowa
Gapdh	Mm.PT.39a.1	Integrated DNA Technologies

SYBR green	
Tnf	forward: CCCTCACACTCAGATCATCTTCT
	reverse: GCTACGACGTGGGCTACAG
Il1b	forward: TGACGGACCCCAAAAGATGA
	reverse: TGCTGCTGCGAGATTTGAAG
Cxcl10	forward: GTGTTGAGATCATTGCCACGA
	reverse: GCGTGGCTTCACTCCAGTTAA
Hmgcr	forward: AGTACATTCTGGGTATTGCTGG
	reverse: ACTCGCTCTAGAAAGGTCAATC
Gapdh	forward: AATGTGTCCGTCGTGGATCTGA
	reverse: GATGCCTGCTTCACCACCTTCT
Il33 promoter	forward: GAAACTCATGCAGACTGTTGACCAA

reverse: GTGCACGATTCTTAGAAAATGTT

PCR primers for mouse genotyping

Gene name	
IL-33	forward: GAGAGATCAAATGAGGCC<br>forward mutant: GGTCGCTACCATTACCAG<br>reverse: GCTGGAGACCAGACTTGT
Kras- LSL-G12D	forward wild-type: GTCGACAAGCTCATGCGGG<br>forward mutant: CCATGGCTTGAGTAAGTCTGC<br>reverse: CGCAGACTGTAGAGCAGCG
Cre	forward: GCATTACCGGTCGATGCAACGAGTGATGAG<br>reverse: GAGTGAACGAACCTGGTCGAAATCAGTGCG
P53 flox	forward: GGTTAAACCCAGCTTGACCA<br>reverse: GGAGGCAGAGACAGTTGGAG
Trif	forward: AGATGGTTCAGCTGGGTGTC<br>reverse wild-type: GGTTCTCCGAACACTCAGTC<br>reverse mutant: GGTTCTCCGAACACTCAGTT
MyD88	forward wild-type: GTTGTGTGTGTCCGACCGT<br>forward mutant: CCACCCTTGATGACCCCCTA<br>reverse: GTCAGAAACAACCACCACCATGC
IRF3	common: GAACCTCGGAGTTATCCCGAAGG<br>wt: GTTTGAGTTATCCCTGCACTTGGG<br>mut: TCGTGCTTTACGCTATCGCCGCTCCCGATT
